# Methodological quality of radiomic-based prognostic studies in gastric cancer: a cross-sectional study

**DOI:** 10.3389/fonc.2023.1161237

**Published:** 2023-09-04

**Authors:** Tianxiang Jiang, Zhou Zhao, Xueting Liu, Chaoyong Shen, Mingchun Mu, Zhaolun Cai, Bo Zhang

**Affiliations:** ^1^ Department of General Surgery, West China Hospital, Sichuan University, Chengdu, China; ^2^ Gastric Cancer Center, West China Hospital, Sichuan University, Chengdu, China; ^3^ Department of Gastrointestinal Cancer Center, Chongqing University Cancer Hospital, Chongqing, China; ^4^ Department of Medical Discipline Construction, West China Hospital, Sichuan University, Chengdu, China

**Keywords:** gastric cancer, radiomics, methodological quality, prognostic, deep learning

## Abstract

**Background:**

Machine learning radiomics models are increasingly being used to predict gastric cancer prognoses. However, the methodological quality of these models has not been evaluated. Therefore, this study aimed to evaluate the methodological quality of radiomics studies in predicting the prognosis of gastric cancer, summarize their methodological characteristics and performance.

**Methods:**

The PubMed and Embase databases were searched for radiomics studies used to predict the prognosis of gastric cancer published in last 5 years. The characteristics of the studies and the performance of the models were extracted from the eligible full texts. The methodological quality, reporting completeness and risk of bias of the included studies were evaluated using the RQS, TRIPOD and PROBAST. The discrimination ability scores of the models were also compared.

**Results:**

Out of 283 identified records, 22 studies met the inclusion criteria. The study endpoints included survival time, treatment response, and recurrence, with reported discriminations ranging between 0.610 and 0.878 in the validation dataset. The mean overall RQS value was 15.32 ± 3.20 (range: 9 to 21). The mean adhered items of the 35 item of TRIPOD checklist was 20.45 ± 1.83. The PROBAST showed all included studies were at high risk of bias.

**Conclusion:**

The current methodological quality of gastric cancer radiomics studies is insufficient. Large and reasonable sample, prospective, multicenter and rigorously designed studies are required to improve the quality of radiomics models for gastric cancer prediction.

**Study registration:**

This protocol was prospectively registered in the Open Science Framework Registry (https://osf.io/ja52b).

## 1 Introduction

Gastric cancer (GC) is the fifth most common cancer and the fourth most common cause of cancer death worldwide (1). Systemic chemotherapy, radiotherapy, surgery, immunotherapy, and targeted therapy have all been shown to be viable treatment options for GC (2–5). However, due to the heterogeneous nature of GC and the high rate of recurrence and metastasis, the current advances in diagnostic techniques and treatment modalities for GC are not yet satisfactory. Current standard treatment strategies often lead to over-treatment with unnecessary toxicity or under-treatment in cases of tumor progression. Therefore, there is an urgent need to develop tools that could be used to clarify the treatment response and prognosis of GC patients before surgery.

**Figure 1:**
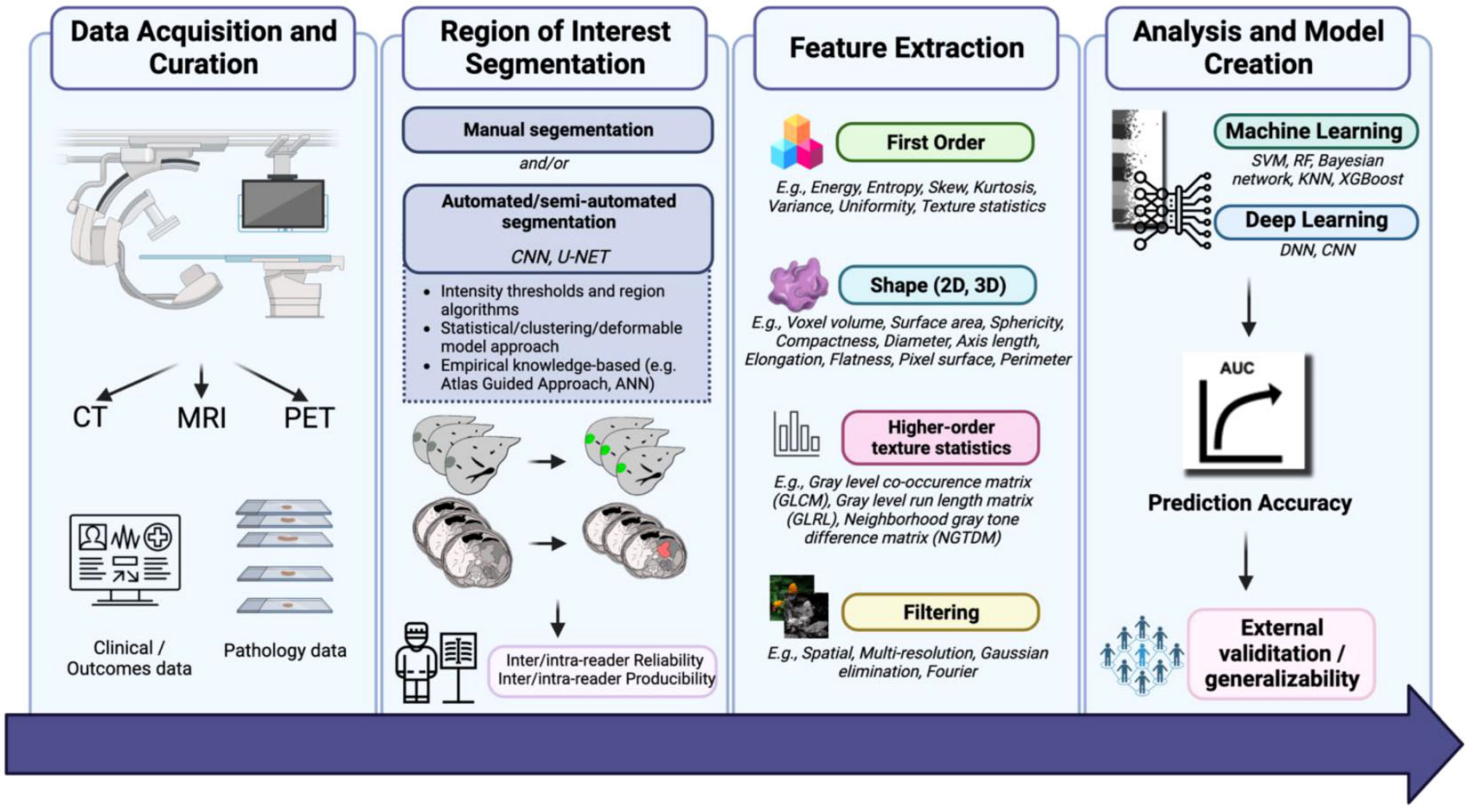
“Flowchart of application of AI in radiology for GI cancers.”, by Azadeh Tabari, licensed under CC BY 4.0.

Radiomics involves the extraction of quantitative metrics (radiomics features) from medical images. This data can be used on its own or combined with demographic, histological, genomic, or proteomic data to build models to solve clinical problems (6). Its main workflow (**Figure 1**) includes data acquisition and curation, region of interest segmentation, feature extraction, analysis and model creation (7). Radiomics is increasingly being used to predict clinical outcomes, particularly in GC (8). However, although numerous studies have evaluated the accuracy of the radiomics model in predicting treatment response in GC, the methodological quality of these studies was not evaluated.

Several tools have been developed to assess the methodological quality of radiomics studies, including the Radiomics Quality Score (RQS) (9), the Transparent Reporting of a multivariable prediction model for Individual Prognosis Or Diagnosis (TRIPOD) (10) assessment tools and the Prediction Risk Of Bias Assessment Tool (PROBAST) (11). The RQS is a standardized assessment tool commonly used to evaluate the scientific integrity and clinical relevance of radiomics oncology studies (12, 13). The TRIPOD tool consists of a checklist designed to evaluate the transparency and completeness of predictive modeling research reports. This tool has been used to evaluate the integrity of numerous oncology radiomics studies (14, 15). The PROBAST was developed to assess the risk of bias and thereby provide a comprehensive evaluation of the methodological quality of primary studies that report predictive model development, validation, or updating (11, 16).

Therefore, this cross-sectional study of the literature aimed to use the RQS, TRIPOD and PROBAST to assess the methodological quality of prognostic radiomics studies related to GC.

## 2 Materials and methods

### 2.1 Eligibility criteria

This study was conducted following the PRISMA guidelines (**Supplementary Material 1**) (17). Due to the rapid advancement in machine learning and radiomics in recent years, only peer-review studies published in last 5 years (between September 2017 and September 2022) were included in this Study. Furthermore, only studies evaluating the prognosis of primary GC in humans based on radiomics features extracted by handcraft or deep learning from clinical images, including computed tomography (CT), magnetic resonance (MR), and positron emission tomography/computed tomography (PET/CT) were included in this study.

Radiomics studies used for diagnostic purposes or to evaluate the degree of differentiation within the tumor were excluded. Studies using models based on non-radiomics features (e.g., standardized uptake values (SUV), clinical parameters, dosimetric parameters, and gene expression data) and those that did not predict prognosis directly were excluded. In addition, case reports, systematic reviews, conference abstracts, editorials, and expert opinion papers were also excluded.

### 2.2 Search methods

The initial literature search was performed using the PubMed and EMBASE electronic databases on 11 September 2022. Since the radiomics studies do not involve randomized controlled studies (RCTs), the Cochrane central database was not searched. Medical Subject Headings (MeSH) and Emtree terms related to GC, radiomics, artificial intelligence, deep learning, and prognosis were used to perform the search. The search strategy is described in more detail in **Supplementary Material 2**.

### 2.3 Selection process

Two researchers (T.J and Z.Z.) searched the PubMed and Embase databases to identify relevant articles. The titles and abstracts of the identified studies were screened independently by the 2 researchers to confirm the eligibility of the studies. Any disagreements in the selection of the studies were resolved via discussion until a consensus was reached. A third researcher (X.L.) was consulted if no consensus was reached. The full texts of the eligible studies were then obtained through an institutional journal subscription and examined by 2 researchers (T.J and Z.Z.) independently for their eligibility based on the criteria described above. The articles that met all the eligibility criteria were included for data extraction and methodological evaluation.

### 2.4 Data extraction

Data extraction was performed independently by two researchers (T.J and Z.Z.) from the included publications. The extracted information comprised general information and methodological characteristics of the studies, including author, year, research design (prospective and retrospective), the number of collaborating institutes, outcome measures, sample size, the radiomics feature extraction method employed (deep learning or handcrafted), the number of features retained in the final model, any additional non-radiomics features used for model development, the performance metrics utilized to assess the model, and the calibration results (if provided).

### 2.5 Analysis of the methodological quality

Two researchers (T.J and Z.Z) evaluated the methodological quality independently using the RQS, TRIPOD and PROBAST. Any disagreements were resolved by consulting with a third researcher (X.L.).

The RQS model proposed by Lambin et al. (9) is based on the steps used to construct a radiomics model and consists of 16 items across 6 domains. The RQS ranges from -8 to 36. The TRIPOD checklist (10) can be used to assess the completeness of the included studies while using RQS (18). This tool consists of 22 main criteria with 37 items. Items 21 and 22 were not evaluated in this study because they assess the supplementary and funding information, respectively. Based on the TRIPOD criteria, the prediction models were classified as development only (type 1a), development and validation using resampling (type 1b), random split sample validation (type 2a), non-random split sample validation (type 2b), validation using separate data (type 3), or validation only (type 4). To assess the risk of bias and applicability of the included studies, PROBAST was employed (16), which includes 20 signaling questions distributed among 4 domains (participants, predictors, outcome, and analysis).

### 2.6 Statistical analysis

The RQS for each item and the total RQS were presented as mean +/- standard deviation (SD). When an item obtained a score of at least 1, it was described as basic adherence. The basic adherence rate was calculated as the percentage number of studies with basic adherence. When an item obtained was higher than the average score, it was considered the ideal score. The percentage number of ideal scores was defined as the number of studies obtaining an ideal score from the total number of studies. The basic adherence rate for TRIPOD was calculated using the same method. TRIPOD item 5c (if completed) and validation items 10c, 10e, 12, 13c, 17, and 19a were excluded from the calculation of the overall adherence rate. The results of PROBAST were summarized as percentages and presented in a visual plot. Signaling question 4.9, “Do predictors and their assigned weights in the final model correspond to the results from the reported multivariable analysis?” was not included as it only applies to regression-based studies. The analyses were conducted using R version 4.2.1.

## 3 Results

### 3.1 Literature search results

**Figure 2:**
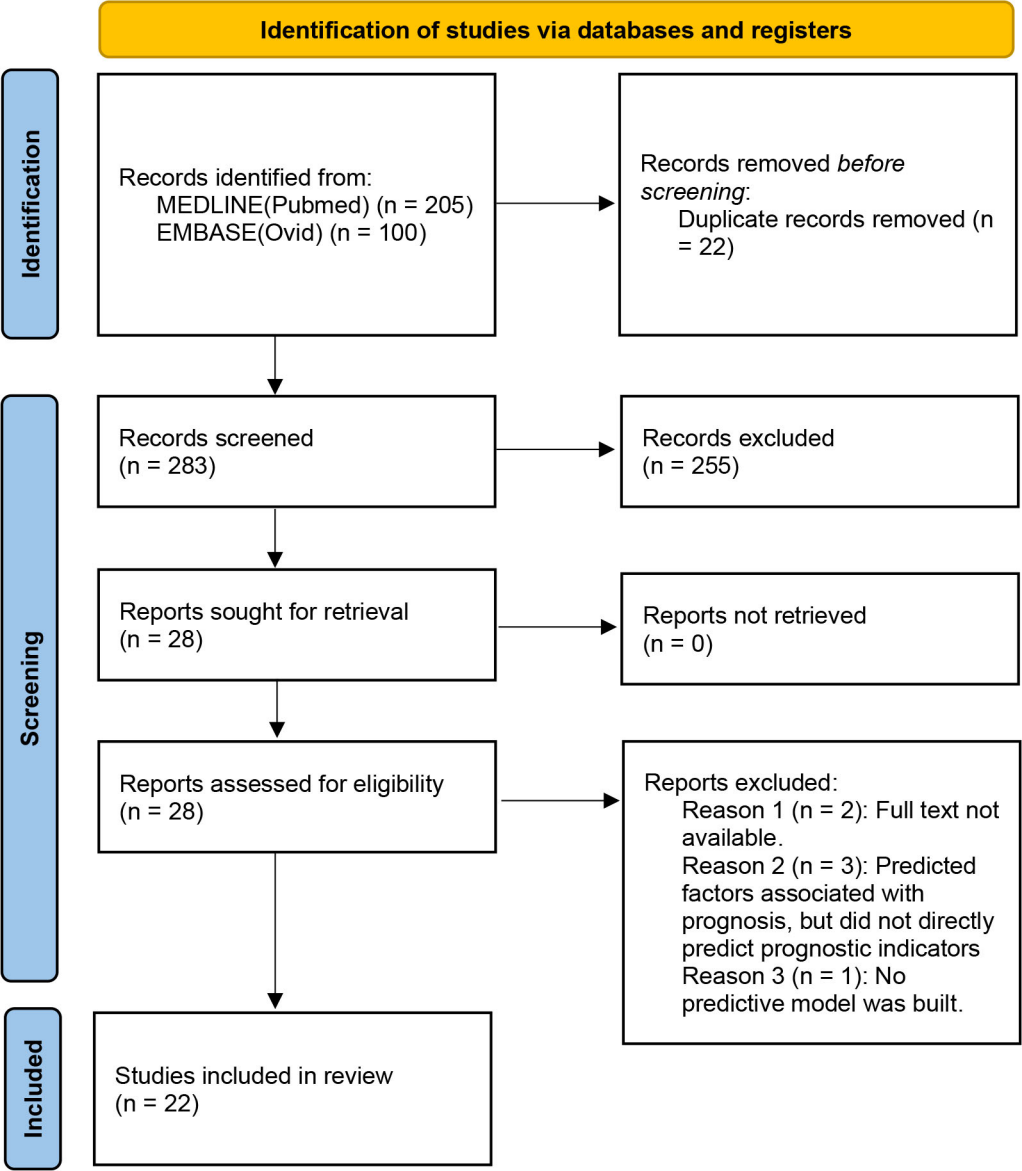
Flowchart of the literature search and study selection (PRISMA 2020).

**Figure 2** illustrates the PRISMA process used to conduct the study. The initial online database search revealed 305 records, of which 205 were retrieved from PubMed, and the rest were retrieved from EMBASE. After removing the 22 duplicates, 283 studies remained for further screening. The screening of the titles and abstracts revealed 28 eligible studies. Six of these studies were excluded after evaluating the full text, and a total of 22 studies (19–40) were finally included in this study.

### 3.2 Basic and methodological characteristics of the included studies

The basic and methodological characteristics of all included studies are summarized in **Table 1**. All studies included in our study were retrospective. Only 8 were multi-institutional, of which 6 included patients from 2 different institutions, and 2 studies included patients from 3 different institutions. Interestingly, almost all of the studies come from Chinese researchers. Some studies did not mention the specific histological type of gastric cancer, while others (5/22) targeted gastric adenocarcinoma. The number of sample size of the included studies ranged from 30 to 2320.

**Table 1:** Basic and methodological characteristics of the included studies.

Study	Data type	# of institution (s)	Country	Cancer type (histology & basal staging)	Sample size	Treatment	Predicted outcome (s)	Type of features	Non-radiomics cofactors	Reported performance	Model calibration test
Jiang, Y, 2018 -1	R	2	China	Gastric adenocarcinoma; T1-4 N0-3 M0-1	228 (train) 186 (internal val) 1177 (external val)	Total or partial radical gastrectomy	OS, DFS	HF	Clinical factors	AUC=0.892 (train) AUC=0.804 (internal val) AUC=0.821 (external val)	Yes
Jiang, Y, 2018 -2	R	1	China	Primary gastric cancer; T1-4 N0-3 M0-1	132 (train) 82 (val)	Total or partial radical gastrectomy	OS, DFS	HF	Clinical factors	AUC=0.796 (train) AUC=0.806 (val)	Yes
Li, Z, 2018	R	1	China	Gastric adenocarcinoma; stage IIA−IIIC: T3−T4 and/or N+	30	Neoadjuvant chemotherapy	GR (TRG 0-1); non-GR (TRG 2-3)	DLF	No	AUC=0.722	No
Li, W, 2019	R	1	China	Gastric cancer; T2-4 N0-3 M0	115 (train) 66 (val)	Radical gastrectomy with D2 lymphadenectomy and R0 resection	OS	HF	Clinical factors	C-index=0.82	Yes
Chen, X, 2020	R	1	China	Gastric cancer; T1-4 N0-3 M0	112 (train) 48 (test)	Radical gastrectomy	LVI, OS, PFS	HF	Clinical factors	(AP+VP) AUC=0.856 (train) AUC=0.792 (test)	Yes
Li, J, 2020-1	R	1	China	Gastric adenocarcinoma; NA	136 (train) 68 (test)	Gastrectomy plus lymph node dissection	OS, PFS	DLF and HF	Clinical factors	AUC=0.84 (train) AUC=0.82 (test)	Yes
Li, J, 2020-2	R	1	China	Gastric cancer; T1-4 N0-3 M0	286 (train) 453 (val)	Radical gastrectomy	DFS	HF	Clinical factors	AUC=0.746 (train) AUC=0.754 (val)	Yes
Sun, K. Y, 2020	R	1	China	Gastric adenocarcinoma; T2-4 N0-3 M0-1	74 (train) 32 (val)	Neoadjuvant chemotherapy (SOX)	responders (TRG 1-2), non-responders (TRG 3-5)	HF	Clinical factors	AUC=0.77 (train) AUC=0.82 (val)	No
Wang, S, 2020	R	2	China	Gastric cancer; T1-4 N0-3 M0	166 (train) 83 (internal val) 104 (external val)	Curative surgery	DFS	HF	Clinical factors	C-index=0.760 (train) C-index=0.720 (internal val) C-index=0.727 (external val)	Yes
Wang, X, 2020	R	1	China	Gastric cancer; T1-3 N0-3 M0	124 (train) 119 (val)	Radical gastrectomy	Poor prognosis, good prognosis	HF	Clinical factors	C-index=0.88	No
Zhang, L, 2020	R	3	China	Gastric cancer; T1-4 N0-3 M0	518 (train) 122 (external val)	Radical gastrectomy	OS	DLF and HF	Clinical factors	C-index=0.82 (train) C-index=0.78 (external val)	Yes
Zhang, W, 2020	R	2	China	Gastric cancer; T2-4 N0-3 M0	302 (train) 219 (internal test) 148 (external test)	Radical gastrectomy with D2 lymphadenectomy and R0 resection	Early recurrence	DLF and HF	Clinical factors	AUC=0.831 (train) AUC=0.826 (internal test) AUC=0.806 (external test)	Yes
Jiang, Y, 2021	R	2	China & America	Gastric cancer; T0-4 N0-3 M0-1	457 (train) 1158 (external val)	Total or partial radical gastrectomy	DFS, OS	DLF and HF	Clinical factors	C-index=0.787 (train) C-index=0.724 (external val)	Yes
Jin, Y, 2021	R	1	China	Primary gastric cancer; T0-4 N0-3 M0-1	172 (train) 245 (val)	Gastrectomy; adjuvant chemotherapy (including S1 alone, XELOX and FOLFIRI/FOLFOX)	DFS, OS	HF	Clinical and genetic factors	AUC=0.965	Yes
Shin, J, 2021	R	2	South Korea	Gastric cancer; T2-4 N0-3 M0	349 (train) 61 (external val)	R0 gastrectomy with D2 lymphadenectomy	RFS	HF	Clinical factors	AUC=0.733 (train) AUC=0.878 (external val)	Yes
Wang, S, 2021	R	1	China	Gastric cancer; T1-4 N0-3 M0	166 (train) 83 (internal val)	Curative surgery	DFS	HF	Clinical factors	C-index=0.707	No
Zhang, L, 2021	R	3	China	Gastric cancer; T1-4a N0-3 M0	337 (train) 181 (val) 122 (test)	Radical gastrectomy	OS	DLF and HF	Clinical factors	C-index=0.77 (train) C-index=0.74 (val) C-index=0.76 (test)	No
Chen, Y, 2022	R	1	China & Germany	Gastric cancer; T3-4a/b Nx M0	104 (train) 52 (test)	Neoadjuvant chemotherapy (SOX); total or partial radical R0 gastrectomy	DFS, OS	HF	Clinical factors	C-index=0.833 (train) C-index=0.810 (test)	Yes
Hao, D, 2022	R	1	America	Gastric cancer; T1-4 N0-3 M0	743 (train) 318 (test)	Total or partial radical gastrectomy	PFS, OS	DLF	Clinical factors	C-index=0.703 (OS) C-index=0.743 (PFS)	No
Jiang, Y, 2022	R	2	China & America	Gastric cancer; T1-4 N0-3 M0	510 (train) 767 (internal val) 1043 (external val)	Gastrectomy	Peritoneal recurrence, DFS	DLF	Clinical factors	C-index=0.654 (train) C-index=0.668 (internal val) C-index=0.610 (external val)	Yes
Liang, Z, 2022	R	1	China	Relapsed or metastatic inoperable gastric adenocarcinoma	58 (train) 29 (val)	PD-1 inhibitor	Non-PD (CR, PR, SD), PD, PFS, OS	HF	Clinical factors and immunohistochemistry	AUC=0.865 (train) AUC=0.778 (val)	Yes
Liu, H, 2022	R	1	China	Gastric cancer; T1-4 N0-3 M0-1	45 (TCGA) 196 (train) 196 (val)	Partial or total radical gastrectomy	OS	HF	Clinical and genetic factors	AUC = 0.838 (train) AUC=0.744 (val)	Yes

R, retrospective; PD-1, programmed cell death 1; LVI, lymphovascular invasion; OS, overall survival; PFS, progression-free survival; GR, good response; non-GR, non-good response; TRG, tumor regression grade; DFS, disease-free survival; RFS, recurrence-free survival; CR, complete remission, PR, partial remission, SD, stable disease; PD, progressive disease; val, validation; HF, handcrafted features; DLF, deep learning-based feature; AUC, area under the receiver operating characteristic curve; C-index, concordance index.

The treatments involved are divided into two types: surgery and medications. Surgery includes partial or total gastrectomy with or without D2 lymphadenectomy. Medications include neoadjuvant chemotherapy or adjuvant chemotherapy with specific regimens such as SOX (S-1 plus oxaliplatin), XELOX (capecitabine plus oxaliplatin), FOLFIRI (folinic acid, fluorouracil, and irinotecan)/FOLFOX (folinic acid, fluorouracil, and oxaliplatin), and a study investigated the impact of PD-1 inhibitors on prognosis of gastric cancer (31).

Different study endpoints were reported in the studies. These were broadly divided into prognosis, treatment response, and other. The prognosis was reported in 18 studies and was artificially classified as poor and good (37) based on overall survival (OS), progression-free survival (PFS), disease-free survival (DFS)/recurrence-free survival (RFS). The pathological treatment response was reported in 3 studies. This category included tumor regression grade (TRG) after neoadjuvant chemotherapy, complete remission (CR), partial remission (PR), stable remission (SR), and progressive remission (PR). The other category included lymphovascular invasion (LVI) (19), early recurrence (40), and peritoneal recurrence (25). The model by Liang et al. was used to predict both the prognosis and treatment response (31).

Seven studies used deep learning models (21, 23, 25, 27, 38–40), and all other studies used only handcrafted features based on Cox proportional hazards, logistic regression (LR), linear regression, support vector machine (SVM) and random forest (RF) models. Most studies (n=21) have combined radiomics features with non-radiomics features (most often clinical factors) to create the models (19–29, 31–40). Of note, the study by Jin et al. used genetic factors (26), and the study by Liang et al. used immunohistochemistry (31). The discriminatory performance of the prognostic prediction model was assessed on the training and validation datasets using either the concordance (C-index) or the area under the curve (AUC). For the training cohort, the C-index ranged from 0.654 (25) to 0.880 (37), and the AUC ranged from 0.722 (30) to 0.965 (26). For the validation cohort, the C-index ranged between 0.610 (25) and 0.810 (20), and the AUC ranged between 0.744 (32) and 0.878 (33).

### 3.3 Assessment of the methodological quality of the studies based on RQS

Based on the steps involved in constructing a radiomics model, the RQS assesses the quality of radiomics studies across 16 projects in 6 key domains. These 6 areas include protocol quality and stability in image and segmentation, feature selection and validation, model performance, biologic/clinical validation and utility, high level of evidence, and open science and data (Details in **Supplementary Table S1**). The overall mean RQS value was 15.32 ± 3.20 (range 9 to 21), which is 42.55% of the ideal 36 scores. Of the 6 domains, domain 5 had the lowest score at 0. Domain 2 achieved the highest mean ideal score (72.16%) of all the six domains. **Table 2** shows the basic adherence rate to the 16 RQS criteria for the 6 domains. The total basic adherence RQS was 59%.

**Table 2:** Radiomics quality score according to the six key domains.

	Basic adherence rate (%)	Mean score	Percentage of the ideal score (%)
Total (ideal score 36)	206 (0.59)	15.32 ± 3.20	42.55
Domain 1: protocol quality and stability in image and segmentation (0 to 5 points)	46 (0.52)	2.09 ± 0.79	41.82
Protocol quality (2 points)	22 (1.00)	1.14 ± 0.34	56.82
Multiple segmentations (1 point)	14 (0.64)	0.64 ± 0.48	63.64
Test–retest (1 point)	10 (0.45)	0.45 ± 0.50	45.45
Phantom study (1 point)	0 (0.00)	0.00 ± 0.00	0.00
Domain 2: feature selection and validation (-8 to 8 points)	44 (1.00)	5.77 ± 1.08	72.16
Feature reduction or adjustment of multiple testing (-3 or 3 points)	22 (1.00)	3.00 ± 0.00	100.00
Validation (-5, 2, 3, 4, or 5 points)	22 (1.00)	2.77 ± 1.08	55.45
Domain 3: model performance index (0 to 5 points)	57 (0.86)	3.36 ± 0.83	67.27
Cut-off analysis (1 point)	19 (0.86)	0.86 ± 0.34	86.36
Discrimination statistics (2 points)	22 (1.00)	1.50 ± 0.50	75.00
Calibration statistics (2 points)	16 (0.73)	1.00 ± 0.74	50.00
Domain 4: biologic/clinical validation and utility (0 to 6 points)	50 (0.57)	3.50 ± 1.62	58.33
Non-radiomics features (1 point)	21 (0.95)	0.95 ± 0.21	95.45
Biologic correlations (1 point)	2 (0.09)	0.09 ± 0.29	9.09
Comparison to ‘gold standard’ (2 points)	13 (0.59)	1.18 ± 0.98	59.09
Potential clinical utility (2 points)	14 (0.64)	1.27 ± 0.96	63.64
Domain 5: high level of evidence (0 to 8 points)	0 (0.00)	0.00 ± 0.00	0.00
Prospective study (7 points)	0 (0.00)	0.00 ± 0.00	0.00
Cost-effectiveness analysis (1 point)	0 (0.00)	0.00 ± 0.00	0.00
Domain 6: open science and data (0 to 4 points)	9 (0.41)	0.59 ± 0.94	14.77

For domain 1, all studies followed the well-documented imaging protocol criteria. Fourteen studies (64%) used multiple segmentation methods (by different physicians/algorithms/software) (19, 21–26, 28, 29, 31, 32, 34, 38, 39), and 10 studies (45%) used images obtained at different time points (19, 20, 24, 27–29, 35, 36, 38, 40). None of the studies conducted phantom studies to assess the feature reliability of the different CT scanners.

For domain 2, all 22 studies conducted feature reduction or adjustment of multiple tests and validation. Fourteen studies (64%) only performed internal validation (19–21, 24, 26–32, 34, 35, 37), and 1 of the studies used the training cohort to validate the model (30). Only 8 studies (36%) were validated using both internal and external datasets (22, 23, 25, 33, 36, 38–40). In one of these studies, 2 external datasets from different centers were used to validate the algorithm (39).

For domain 3, 19 studies (86%) made use of cut-off analysis (19, 21–27, 30–40). All 22 studies used the AUC of a receiver operating characteristic curve for discrimination analysis, and 16 studies (73%) used calibration statistics (19, 20, 22–29, 31–33, 36, 38, 40).

For domain 4, multivariate analysis of non-radiomics features was performed in almost all studies (n=21, 95%). Biological correlations were involved in 2 (9%) studies (26, 32). The performance of the radiomics models was assessed by comparing the results with “gold standards” in 13 (59%) studies (19–25, 27, 31, 32, 35, 36, 39). The potential clinical utility of the model was assessed in 14 studies (19, 20, 22–26, 28, 29, 31, 32, 34, 36, 38).

For domain 5, none of the included studies were prospective. Furthermore, no studies conducted a cost-effectiveness analysis.

For domain 6, 9 studies (41%) (20, 21, 23, 25, 26, 31–33, 39) used an open-source code to develop the model.

### 3.4 Analysis of reporting completeness based on the TRIPOD checklist

In order to increase the transparency of research reports on predictive modeling, the TRIPOD statement has developed a checklist in 5 areas: title and abstract, introduction, methods, results, and discussion. The reporting completeness of the included studies according to the TRIPOD checklist is summarized in **Table 3** and **Supplementary Table S2**. After excluding both the “if done” in item 5c and the validation items 10c, 10e, 12, 13c, 17, and 19a from the numerator and denominator, the mean number of adherences with the 35 items on the TRIPOD checklist was 20.45 ± 1.83, and the adherence rate was 73.05% ± 6.53%.

**Table 3:** Adherence to individual TRIPOD items in radiomics studies.

	All articles (n =22)
Total (35 items)	
Title and abstract	
1. Title: identify developing/validating a model, target population, and the outcome	22 (100)
2. Abstract: provide a summary of objectives, study design, setting, participants, sample size, predictors, outcome, statistical analysis, results, and conclusions	18 (81.82)
Introduction	
3a. Explain the medical context and rationale for developing/validating the model	22 (100)
3b. Specify the objectives, including whether the study describes the development/validation of the model or both	22 (100)
Methods	
4a. Source of data: describe the study design or source of data (randomized trial, cohort, or registry data)	22 (100)
4b. Source of data: specify the key dates	21 (95.45)
5a. Participants: specify key elements of the study setting including number and location of centers	22 (100)
5b. Participants: describe eligibility criteria for participants (inclusion and exclusion criteria)	21 (95.45)
5c. Participants: give details of treatment received, if relevant (n = 4)	4 (18.18)
6a. Outcome: clearly define the outcome, including how and when assessed	17 (77.27)
6b. Outcome: report any actions to blind assessment of the outcome	3 (13.64)
7a. Predictors: clearly define all predictors, including how and when assessed	17 (77.27)
7b. Predictors: report any actions to blind assessment of predictors for the outcome and other predictors	0 (0)
8. Sample size: explain how the study size was arrived at	1 (4.55)
9. Missing data: describe how missing data were handled with details of any imputation method	2 (9.09)
10a. Statistical analysis methods: describe how predictors were handled	22 (100)
10b. Statistical analysis methods: specify type of model, all model-building procedures (any predictor selection), and method for internal validation	21 (95.45)
10d. Statistical analysis methods: specify all measures used to assess model performance and if relevant, to compare multiple models (discrimination and calibration)	20 (90.91)
11. Risk groups: provide details on how risk groups were created, if done (yes or no, n = 14)	14 (63.64)
Results	
13a. Participants: describe the flow of participants, including the number of participants with and without the outcome. A diagram may be helpful	14 (63.64)
13b. Participants: describe the characteristics of the participants, including the number of participants with missing data for predictors and outcome	21 (95.45)
14a. Model development: specify the number of participants and outcome events in each analysis	22 (100)
14b. Model development: report the unadjusted association between each candidate predictor and outcome, if done (yes or no, n = 1)	1 (4.55)
15a. Model specification: present the full prediction model to allow predictions for individuals (regression coefficients, intercept)	3 (13.64)
15b. Model specification: explain how to the use the prediction model (nomogram, calculator, etc.)	17 (77.27)
16. Model performance: report performance measures (with confidence intervals) for the prediction model	22 (100)
Discussion	
18. Limitations: Discuss any limitations of the study	21 (95.45)
19b. Interpretation: Give an overall interpretation of the results	21 (95.45)
20. Implications: Discuss the potential clinical use of the model and implications for future research	21 (95.45)
For validation (types 2a, 2b, 3, and 4)	
10c. Methods-Statistical analysis methods: describe how the predictions were calculated	14 (63.64)
10e. Methods-Statistical analysis methods: describe any model updating (recalibration), if done	1 (4.55)
12. Methods-Identify any differences from the development data in setting, eligibility criteria, outcome, and predictors	0 (0)
13c. Results-show a comparison with the development data of the distribution of important variables	17 (77.27)
17. Results-Model updating: report the results from any model updating, if done	1 (4.55)
19a. Discussion-Interpretation: discuss the results with reference to performance in the development data and any other validation data	21 (95.45)

**Figure 3:**
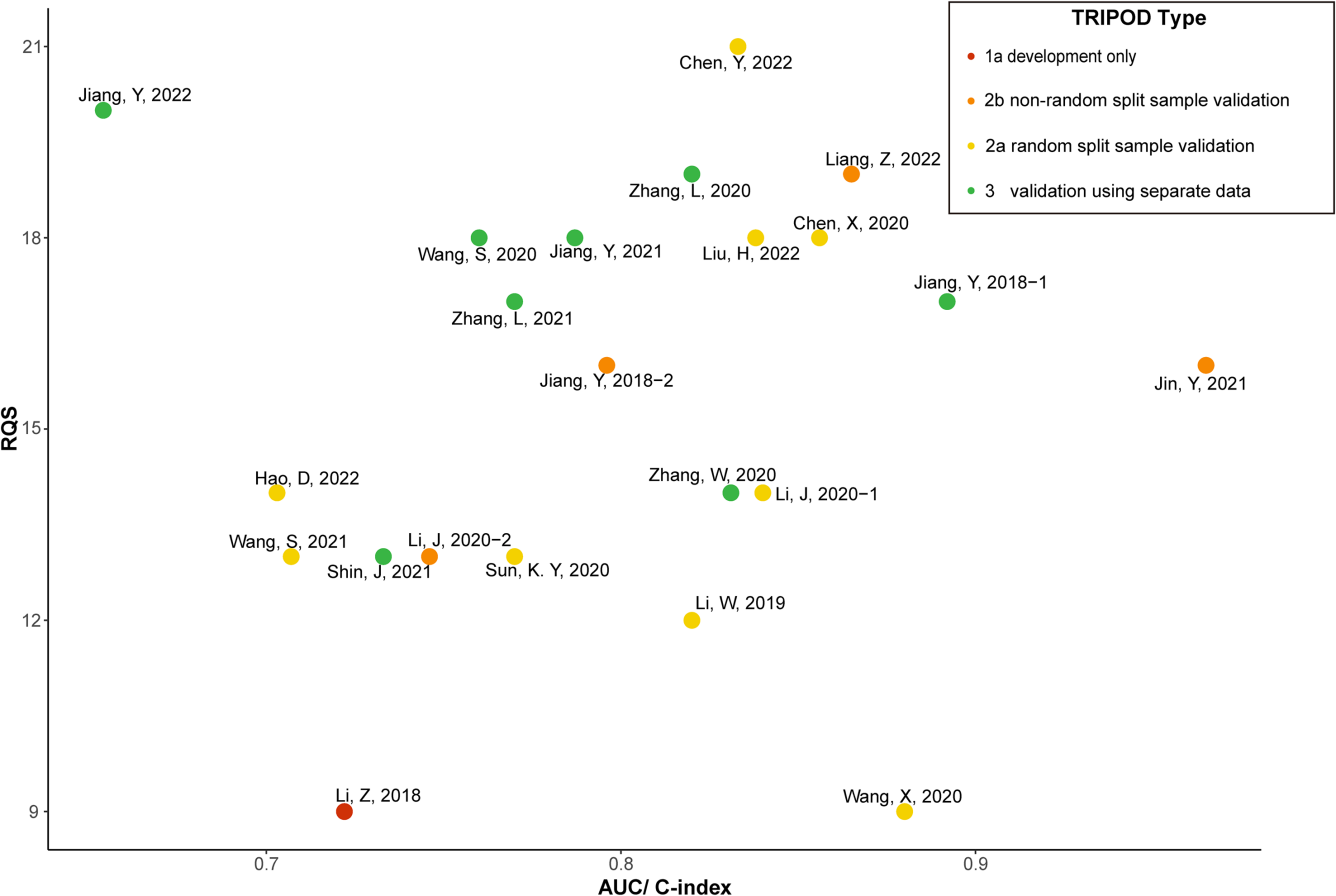
The AUC/C-index and RQS reported by the included studies classified by TRIPOD.

**Figure 3** shows the AUC/C-index and RQS reported by the included studies classified by TRIPOD. The different TRIPOD classifications are illustrated using different colors. The studies with the higher RQS had a better TRIPOD classification [usually 2a (19, 32) or 3 (22, 23, 36, 38, 39)]. Furthermore, these studies also had a higher AUC or C-index ranging from 0.760 (36) to 0.892 (22).

### 3.5 Assessment of the risk of bias based on PROBAST

The risk of bias assessment based on PROBAST is summarized in **Table 4** and **Supplementary Table S3**. Almost all studies (95.45%) were classified as low risk in the participant domain, except for one study that did not mention the inclusion and exclusion criteria for participants (35). In the predictors domain, half of the studies (50%) were rated as high risk due to the lack of blinding in accessing predictors (19, 24, 25, 27, 29, 31, 33, 35, 36, 38, 39). All studies were rated as low risk in the outcome domain. However, in the analysis domain, only one study (36) was rated as low risk, while most studies (95.45%) were rated as high risk due to do not perform reasonable sample size estimating (19–35, 37–40). In addition, some studies did not provide information on the handling of continuous and categorical predictors (27.27%) (21, 27, 32, 33, 35, 39) and participants with missing data (86.36%) (19, 21–35, 38–40).

**Table 4:** PROBAST signaling questions in 22 included studies.

Signaling question	Yes or probably yes (%)	No or probably no (%)	No information (%)
Participants domain			
1.1. Were appropriate data sources used, for example, cohort, randomized controlled trial, or nested case-control study data?	22 (100)	0	0
1.2. Were all inclusions and exclusions of participants appropriate?	21 (95.45)	1 (4.55)	0
Predictors domain			
2.1. Were predictors defined and assessed in a similar way for all participants?	22 (100)	0	0
2.2. Were predictor assessments made without knowledge of outcome data?	11 (50.00)	11(50.00)	0
2.3. Are all predictors available at the time the model is intended to be used?	22 (100)	0	0
Outcome domain			
3.1. Was the outcome determined appropriately?	22 (100)	0	0
3.2. Was a prespecified or standard outcome definition used?	22 (100)	0	0
3.3. Were predictors excluded from the outcome definition?	22 (100)	0	0
3.4. Was the outcome defined and determined in a similar way for all participants?	22 (100)	0	0
3.5. Was the outcome determined without knowledge of predictor information?	22 (100)	0	0
3.6. Was the time interval between predictor assessment and outcome determination appropriate?	22 (100)	0	0
Analysis domain			
4.1. Were there a reasonable number of participants with the outcome?	1 (4.55)	21 (95.45)	0
4.2. Were continuous and categorical predictors handled appropriately?	16 (72.73)	0	6 (27.27)
4.3. Were all enrolled participants included in the analysis?	21 (95.45)	1 (4.55)	0
4.4. Were participants with missing data handled appropriately?	3 (13.64)	0	19 (86.36)
4.5. Was selection of predictors based on univariable analysis avoided? (Development studies only)	21 (95.45)	1 (4.55)	0
4.6. Were complexities in the data (e.g., censoring, competing risks, sampling of control participants) accounted for appropriately?	2 (9.09)	1 (4.55)	19 (86.36)
4.7. Were relevant model performance measures evaluated appropriately?	21 (95.45)	1 (4.55)	0
4.8. Were model overfitting, underfitting, and optimism in model performance accounted for? (Development studies only)	22 (100)	0	0

Signaling question 4.9 “Do predictors and their assigned weights in the final model correspond to the results from the reported multivariable analysis?” was not included as it applies to regression-based studies.

PROBAST, prediction risk of bias assessment tool; NA, not applicable to external validation.

**Figure 4:**
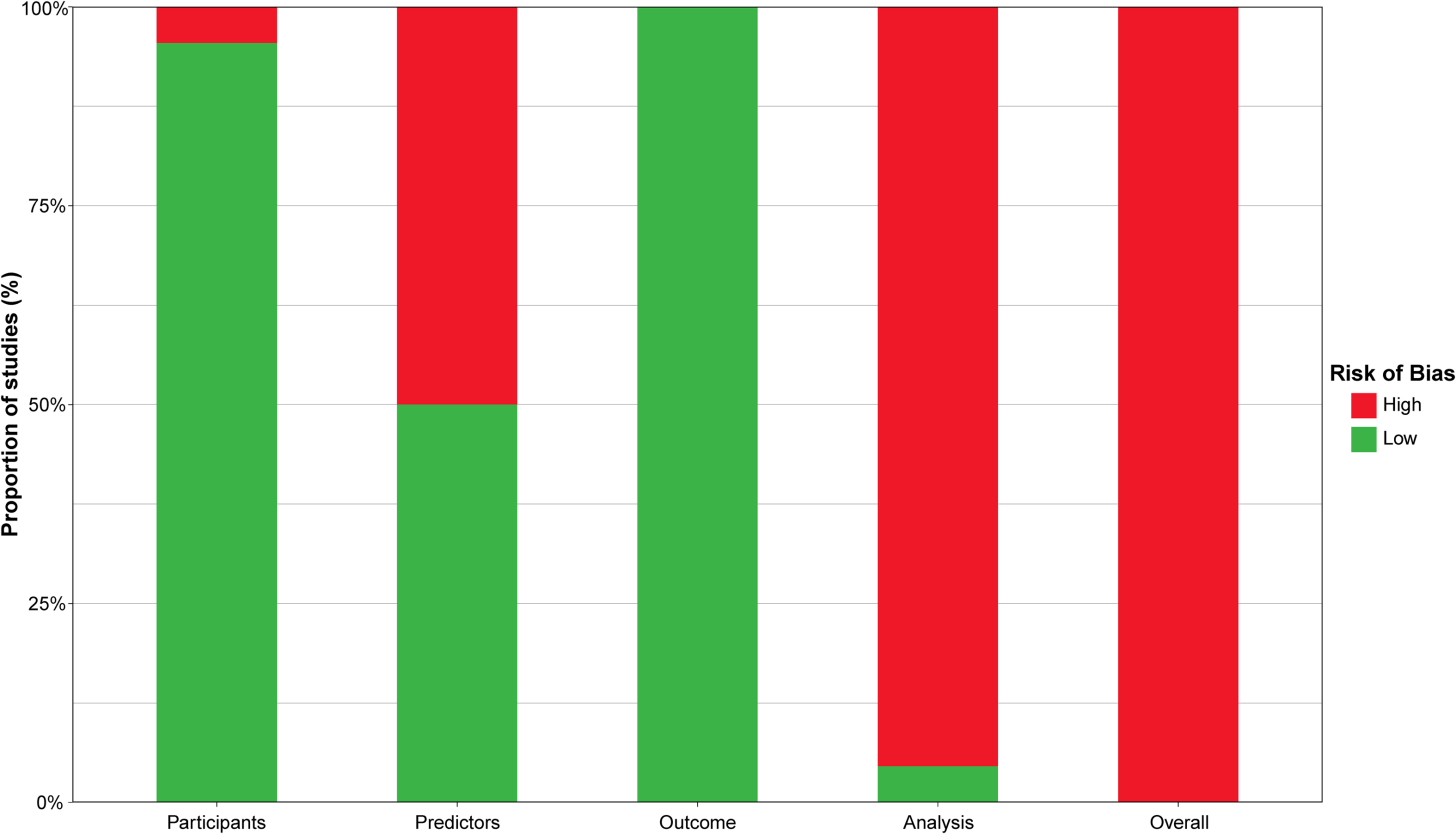
Risk of bias of included studies.

All studies had at least one high-risk domain, with participants and analysis sections being the most frequent. Therefore, all studies were ultimately rated as high risk of bias. The four domains and the overall risk of bias of the included studies are visualized in **Figure 4**.

## 4 Discussion

This study aimed to assess the methodological characteristics and quality of radiomics studies predicting the prognosis of patients with GC published in the last 5 years, using RQS, TRIPOD, and PROBAST. All studies included in this study were retrospective, which may have introduced inaccuracies in prognosis-focused follow-up. Furthermore, the included studies mainly focused on the prognosis of GC patients after gastrectomy, with only a few studies evaluating the prognosis after adjuvant chemotherapy, neoadjuvant chemotherapy, or PD-1 inhibitor therapy. Additionally, the sample size of most studies was insufficient for building stable predictive models and lacked reasonable sample size estimation in advance. Clinical factors were incorporated in almost all currently available radiomics prognostic models for GC, with some models also incorporating genetic factors (26, 32) or immunohistochemistry (31). The integration of radiomics with clinical and genetic features has been shown to improve the predictive performance of prognostic models (41). However, most studies did not perform external validation, potentially limiting the generalizability of the models. The lack of standardized practices for analyzing radiomics models, limited data sharing between institutions, and the lack of automated segmentation are currently limiting the adoption of these models in prospective clinical studies (42). Thus, further prospective multicenter studies with larger and adequately powered samples are necessary to improve the quality and generalizability of prognostic radiomics models for GC.

Upon evaluating the radiomics prognostic prediction models for GC using the RQS, our study revealed a lack of scientific quality in the current models, particularly in domain 1, domain 5, and domain 6. Notably, none of the included studies conducted a phantom study on all scanners, despite previous research showing that the variability of the values of radiomic features calculated on CT images from different CT scanners can be comparable to the variability of these features found in CT images of other tumor (43). Consequently, future radiomic studies in gastric cancer should consider and minimize the impact of differences between scanners. Additionally, none of the included studies met the high level of evidence criteria, as all were retrospective and lacked cost-effectiveness analyses. Our analysis also showed low scores in biologic correlations (9.09%) and open science/data (14.77%), which are similar to the limitations observed in radiomics models used for other purposes (44–46).

Upon assessment of reporting completeness using the TRIPOD checklist, the included studies showed poor basic adherence rates, particularly for items such as blinding when assessing results, demonstrating how the required sample size was reached, handling of missing data, and presenting the entire prediction model. These results are consistent with previous reviews on radiomics and oncology studies that also utilized TRIPOD (45–47). Therefore, there is a pressing need to address these aspects to ensure that reporting of prognostic GC radiomics prediction models is more transparent, complete, and standardized. It should be noted, however, that the current TRIPOD checklist is mainly focused on regression-based predictive model approaches, limiting its applicability to artificial intelligence and machine learning research, which typically do not require regression analysis. To address this limitation, a new version of the TRIPOD statement for machine learning is currently in development (48).

The evaluation of included studies using PROBAST revealed that all of them were at high risk of bias. Contributing factors to bias included a failure to use blinding to obtain predictors, a lack of reasonable sample size estimation in advance, and improper handling of participants with missing data. Similarly, most of the radiomics studies examining other diseases were also found to be at high risk of bias. A systematic review of radiomic prognostic prediction models for breast cancer showed that 95.7% of the included studies were at high risk of bias (49). Similarly, a systematic review of radiomic prognostic prediction studies for non-small cell lung cancer found that all of the included studies were at high risk of bias (50). Furthermore, these reviews also identified participant and analytic domains as the primary sources of bias.

This study has some limitations that have to be acknowledged. Due to the small number of existing studies and the wide range of mathematical tools used to assess the performance of the models, it was not possible to perform a quantitative meta-analysis. In addition, several items on the RQS and TRIPOD tools could not be assessed as they do not apply to prognostic radiomics models. It is also important to acknowledge that some items on the RQS are over-idealistic and are difficult to achieve in practice (51). Furthermore, the TRIPOD checklist was designed to facilitate the reporting of radiomics studies and not to assess the methodological quality of radiomics studies (52). Finally, although we did our best to use objective criteria, independent raters, and dissent negotiations to evaluate the methodological quality of the radiomics studies, there may still be some unavoidable bias in our evaluation. We searched for worldwide studies in this area and found that the main country of publication was China, which may lead to geographical bias and may not have broad extrapolation.

Our findings indicate that the current methodological quality of radiomics studies for prognosis prediction in GC is insufficient. Therefore, larger and reasonable sample size, prospective, multicenter, and rigorously designed studies are required to improve the generalizability of the models. Future radiomics studies should also include phantom studies on the scanners, more biological correlations, and open science/data.

## Data availability statement

The original contributions presented in the study are included in the article/**Supplementary Material**. Further inquiries can be directed to the corresponding authors.

## Author contributions

BZ and ZC contributed to conception and design of the study. TJ, ZZ and XL collected the data. CS and MM performed the statistical analysis. TJ wrote the first draft of the manuscript. TJ, ZZ and XL wrote sections of the manuscript. All authors contributed to manuscript revision, read, and approved the submitted version.
