# Supplementary material for: Methodological quality of radiomic-based prognostic studies in gastric cancer: a cross-sectional study
Source: Front Oncol. 2023 Sep 4;13:1161237. doi: 10.3389/fonc.2023.1161237 (PMC10507631; doi:10.3389/fonc.2023.1161237)
Supplement: Supplementary file 2 [file DataSheet_2.docx]

1. **PubMed Medline**

"Stomach Neoplasms"[MeSH Terms] AND ("radiomic"[All Fields] OR "radiomics"[All Fields] OR "Deep Learning"[MeSH Terms] OR "Artificial Intelligence"[MeSH Terms] OR "machine learning"[All Fields] OR "automation"[All Fields]) AND "Prognosis"[MeSH Terms]

1. **Embase: the search terms, search steps and number of studies retrieved.**

1 exp stomach cancer/ 140215

2 exp radiomics/ 5703

3 exp artificial intelligence/ 65917

4 exp deep learning/ 28746

5 exp prognosis/ 853624

6 2 or 3 or 4 93683

7 1 and 5 and 6 100
